# Health Benefits and Participation Barriers of Different Level Horseback Riders Age-Wise

**DOI:** 10.3389/fpsyg.2022.889605

**Published:** 2022-04-28

**Authors:** Iker Sáez, Patxi León-Guereño, Ewa Malchrowicz-Mośko, Eneko Balerdi, Xabier Río, Beñat Lavín, Josu Solabarrieta

**Affiliations:** ^1^Department of Physical Activity and Sport Science, Faculty of Education and Sport, University of Deusto, Bilbao, Spain; ^2^Department of Physical Activity and Sport Science, Faculty of Education and Sport, University of Deusto, Donostia-San Sebastian, Spain; ^3^Faculty of Sport Sciences, Eugeniusz Piasecki University of Physical Education, Poznań﻿, Poland; ^4^Department of Educational Innovation and Organization, Faculty of Education and Sport, University of Deusto, Bilbao, Spain

**Keywords:** health benefits, physical inactivity, physical activity, horseback riding, barriers, perceived benefits and barriers

## Abstract

Although horseback riding is not specifically mentioned in the recommendations for different age groups regarding the level of physical activity necessary for good health, its practice continues to grow in popularity throughout the world. Despite being a minority discipline, it has some characteristics that make it an opportunity for its participants to be active people, so it is important to understand what are the perceived health benefits and barriers to participation. The aim of the study is to describe and analyze the perceived health benefits and barriers in horseback riding among riders categorized by level and age, in order to promote physical activity through these benefits and to overcome the barriers. An online version of the EBBS (Exercise Benefits/Barriers Scale) was used to analyze benefits and barriers. The sample consisted of 2,651 participants (95.9% women and 4.1% men) in an equestrian event, distributed in four age groups (79.4% up to 25 years, 11.5% between 26 and 35, 7.9% between 36 and 50, and 1.2 older than 50 years). Perceived benefits and participation barriers to horseback riding were analyzed. The factor analysis identified and confirmed five benefit factors and four barrier factors. Benefit factors were significantly correlated among them but barriers were less interrelated. Higher ages were associated to larger benefits and less barrier effects. Benefit and barrier differences were larger between amateur and professional riders, compared to gender differences.

## Introduction

The benefits of having a physically active lifestyle during different stages of life are well documented (Guthold et al., 2020). Recent systematic reviews (Crane and Temple, 2015; Eime et al., 2015) have shown that physical activity contributes to the improvement of the physical and mental state and as a factor of protection, promotion, and maintenance of health, wellbeing, and quality of life by helping to reduce the stress and improving the cognition, thinking skills, and strengthen functional abilities (Martínez-Heredia et al., 2021). Regular exercise has been confirmed to counteract fragility and sarcopenia; reduce the risk of many chronic diseases; reduce the incidence of depression and dementia; and improve general wellbeing (López-Sanchez et al., 2016; Marzetti et al., 2017; Simas et al., 2017). Given its significance, the World Health Organization (WHO) offers guidelines to achieve these benefits. The WHO recommends to accumulate a minimum of 150–300 min of moderate-intensity aerobic physical activity, or a minimum of 75–150 min of vigorous-intensity aerobic physical activity, or an equivalent combination of moderate- and vigorous-intensity activities, during the week in order to obtain significant health benefits (World Health Organization, 2020). Physical activity can be defined as any type of muscular activity that substantially increases energy consumption (Shephard, 2003).

Higher levels of sedentary behavior are associated with higher mortality (Patterson et al., 2018; Ekelund et al., 2019). Individuals who do not fulfill the recommendations for moderate and/or vigorous activity are considered to be inadequately active or inactive (Guthold et al., 2018). Despite extensive evidence of the numerous benefits of physical activity, recent surveys show that many people do not follow these recommendations (Hallal et al., 2012; Antoniewicz and Brand, 2016). Recent estimates indicate that in European countries, approximately 60% of the population never or hardly ever engage in sports and more than half of the population engage in regular physical activity (walking, cycling, stair climbing…; Sarkar and Fletcher, 2014). Poland in particular, as in other societies in the Central European region, is undergoing a social, economic, and moral transition, which is causing fast and deep changes in the lifestyle of its citizens, including: alcohol abuse, inadequate dietary patterns, tobacco consumption, and reduced levels of physical activity that result in a key problem in the prevention and control of non-communicable diseases (Drygas et al., 2009).

One of the reasons for physical inactivity is people’s perceived barriers to physical activity (Fernández and Ropero, 2015). Perceived barriers are defined as barriers that make it difficult to engage in behavior such as physical exercise (Ramírez-Vélez et al., 2016). Perceived barriers to physical activity have been shown to have a negative correlation with the perceived benefits of physical activity (VanZanten et al., 2015). Thus, the analysis of barriers is very important not only to be able to avoid them, but also because perceived barriers are associated with a higher prevalence of physical inactivity (Dias et al., 2015). Identifying barriers and educating on how to overcome them can be a key component of successfully increasing physical activity (Kulavic et al., 2013).

Recreational horseback riding is not specifically mentioned within the physical activity recommendations, although owning a horse will result in some activity (Machová et al., 2019). As a leisure activity, it provides the opportunity to achieve the recommended objectives of physical activity levels and has been identified as one of several “green exercises” (activities involving contact with the natural environment and green spaces; Pretty et al., 2007). Despite the growth in popularity of horseback riding, the scientific literature has focused on studying the benefits of horseback riding for people with disabilities (MacKinnon et al., 1995); few studies have examined the benefits and barriers for riders without such disorders. Given such growth, it is important to understand the motivations, benefits, and perceived barriers to this activity in order to understand and promote this type of physical activity (Burbage and Cameron, 2018).

Horseback riding is more than a physical or leisure activity, it is a real therapy with beneficial effects on health, understood in a global way (Stergiou et al., 2017). Different research carried out in the United Kingdom analyzed horseback riding and found that it is a medium to high intensity exercise (Beale et al., 2015). The regular practice of horseback riding is associated with physical, social, and psychological health benefits and improved wellbeing (Maxwell et al., 2011). Balance, coordination, and posture are improved, better reflexes, muscle development, etc. (Koca, 2016). However, and despite the multiple benefits of this activity, the participation barriers still remain unclear. Therefore, the aim of the study is to describe and analyze the perceived health benefits and barriers in horseback riding among riders categorized by level and age, in order to promote physical activity through these benefits and to overcome the barriers.

## Materials and Methods

### Subjects and Design

An online questionnaire was used to carry out the research. Participants were contacted during an equestrian event, with the agreement of the event organizer. As online surveys or questionnaires do not require the completion of a physical informed consent, it was reported that the completion of the form constituted informed consent. The survey was anonymous, voluntary, and confidential. This is a descriptive, quantitative, and cross-sectional research, whose sample consisted of 2,651 participants in the equestrian event. Participants were distributed in four age groups (79.4% up to 25 years, 11.5% between 26 and 35, 7.9% between 36 and 50, and 1.2 older than 50 years). Out of the total, 2,651 (95.9%) were women and 111 (4.1%) were men.

The research was carried out in accordance with the Helsinki Declaration of 1975, and the study was treated in accordance with the guidelines of the Publication Manual of the American Psychological Association regarding consent and anonymity. The questionnaire was created using Google Docs technology and it was voluntary, anonymous, and confidential. In Poland, anonymous diagnostic surveys do not require approval by a bioethics committee.

### Instruments

Literature review has revealed that the Exercise Benefits/Barriers Scale (EBBS; Sechrist et al., 1987) is the most widely used instrument to measure the benefits and barriers to physical activity. The EBBS is composed of 43 items presented on a Likert-type scale with four response possibilities ranging from four (strongly agree) to one (strongly disagree); 29 of them are related to benefits and 14 to barriers. When assessing the use of the instrument, its validation was consulted and it was considered that at the time of validation, an internal consistency of a Cronbach’s standardized alpha of 0.952 was obtained. Benefits are classified into five sub-scales: life improvement, physical performance, psychological outlook, social interaction, and preventive health. Barriers are subdivided into four sub-scales: exercise-related environment, time investment for exercise, physical effort, and family discouragement.

### Statistical Analysis

The analyses consisted of frequency distributions, univariate descriptive analyses, and bivariate statistical analyses, such as *t*-test, analysis of variance, and correlations. The hypothesis testing used a significance level of 0.05. Statistical analyses were carried out using SPSS (v. 28) and Amos (v. 28).

The measurement model was analyzed combining a Principal Component Analysis and a Confirmatory Factor Analysis. We divided the sample into two halves, randomly assigning each case to one of these. In order to identify the underlying model in the responses using an exploratory technique in one part of the sample and check the model’s adjustment of the resulting measurement in the other, we successively carried out a Principal Component Analysis with the first half (*n* = 1,326) and a Confirmatory Factor Analysis with the second (*n* = 1,325). The factors were extracted using Principal Component Analysis and rotated using the Varimax method. The Confirmatory Factor Analysis included calculations by bootstrapping, as well as the calculation of goodness-of-fit indexes, such as the Root Mean Square Error of Approximation (RMSEA), the Standardized Root Mean Square Residual (SRMR), and the Comparative Fit Index (CFI).

## Results

In the exploratory stage (Tables 1, 2), questions about benefits and barriers were analyzed in two separated Principal Component Analysis. Kaiser-Meyer-Olkin test results were 0.940 for benefit factors and 0.718 for barriers, and Bartlett’s sphericity test value of *p* was 0.000 in both. The analysis identified five benefit components [(1) physical, (2) psycho-social capacities, (3) relaxation, (4) cardiovascular (preventive health), and (5) social contact] and five barrier components [(1) time, (2) fatigue, (3) lack of facilities, (4) family, and (5) embarrassment].

**Table 1:** Benefit indicators’ principal component analysis.

	1	2	3	4	5
15. Horseback riding increases my level of physical fitness.	0.818	0.145	0.136	0.126	0.116
17. My muscle tone is improved with horseback riding.	0.816	0.142	0.151	0.230	
7. Horseback riding increases my muscle strength.	0.815		0.107	0.124	
22. Horseback riding increases my stamina.	0.779	0.225	0.206	0.104	0.108
31. My physical endurance is improved by horseback riding.	0.735	0.274	0.217		0.200
23. Horseback riding improves my flexibility.	0.610	0.312	0.217	0.146	
43. Horseback riding improves the way my body looks.	0.581	0.418	0.121		
8. Horseback riding gives me a sense of personal accomplishment (discarded).	0.474	0.126			0.378
36. Horseback riding improves the quality of my work.	0.205	0.743	0.143	0.134	
35. Horseback riding allows me to carry out normal activities without becoming tired.	0.399	0.638			0.134
34. Horseback riding increases my mental alertness.	0.393	0.594			0.138
27. I will live longer if I ride.	0.203	0.592	0.275	0.245	
26. Horseback riding helps me sleep better at night.	0.183	0.570	0.273	0.173	
32. Horseback riding improves my self-concept.	0.256	0.553	0.274		0.293
39. Horseback riding increases my acceptance by others (discarded).		0.543			0.431
41. Horseback riding improves overall body functioning for me.	0.469	0.518	0.278	0.144	0.103
29. Horseback riding helps me decrease fatigue (discarded).		0.468	0.420	0.231	
10. Horseback riding makes me feel relaxed.	0.137	0.154	0.793		
25. My disposition is improved with horseback riding.	0.260	0.195	0.736		0.110
2. Horseback riding decreases feelings of stress and tension for me.		0.199	0.728		−0.108
3. Horseback riding improves my mental health.	0.164	0.260	0.723		
1. I enjoy horseback riding.	0.149		0.700		0.158
38. Horseback riding is good entertainment for me (discarded).			0.502		0.262
20. I have improved feelings of wellbeing from horseback riding (discarded).	0.310	0.250	0.435	0.119	0.134
13. Horseback riding will keep me from having high blood pressure.	0.196	0.176		0.889	
5. I will prevent heart attacks by horseback riding.	0.157	0.195		0.877	
18. Horseback riding improves functioning of my cardiovascular system.	0.475	0.236	0.101	0.624	
30. Horseback riding is a good way for me to meet new people.	0.169	0.218			0.810
11. Horseback riding lets me have contact with friends and persons I enjoy.	0.166		0.143		0.796

*Rotated component matrix*.

**Table 2:** Barriers indicators’ Principal Component Analysis.

	1	2	3	4	5
24. Horseback riding takes too much time from family relationships.	0.863				
4. Horseback riding takes too much of my time.	0.811				
37. Horseback riding takes too much time from my family responsibilities.	0.744	0.149	0.108	0.122	
14. It costs too much to horseback riding (discarded) (discarded).	0.431	0.252	0.428	0.145	
6. Horseback riding tires me.		0.874			
19. I am fatigued by horseback riding.		0.833			0.167
40. Horseback riding is hard work for me (discarded).	0.329	0.574			
9. Places for me to horseback riding are too far away.			0.802		
42. There are too few places for me to horseback riding.			0.776		
16. Horseback riding facilities do not have convenient schedules for me (discarded).			0.558	0.126	0.329
21. My spouse (or significant other) does not encourage horseback riding.				0.856	
33. My family members do not encourage me to ride.			0.128	0.846	
12. I am too embarrassed to horseback riding (discarded).					0.781
28. I think people in horseback riding clothes look funny (discarded).			0.109		0.766

*Rotated component matrix*.

Several items were discarded because of its cross-loading in more than one factor or its low weight on it: benefits’ item numbers 8, 20, 29, 38, and 39, and barriers’ item numbers 12, 14, 16, 28, and 40. All the resulting factors showed adequate internal consistency indices (Table 3). The only exception was factor 5, which was also discarded for having a coefficient below 0.5.

**Table 3:** Internal consistency indices (Cronbach’s alpha).

Factor	Number of items	Cronbach’s alpha
BE1—Physical	7	0.907
BE2—Psycho-social capacities	7	0.850
BE3—Relaxation	5	0.841
BE4—Cardiovascular	3	0.856
BE5—Social contact	2	0.754
BA1—Time	3	0.773
BA2—Fatigue	2	0.759
BA3—Facilities	2	0.599
BA4—Family	2	0.666

**Figure 1:**
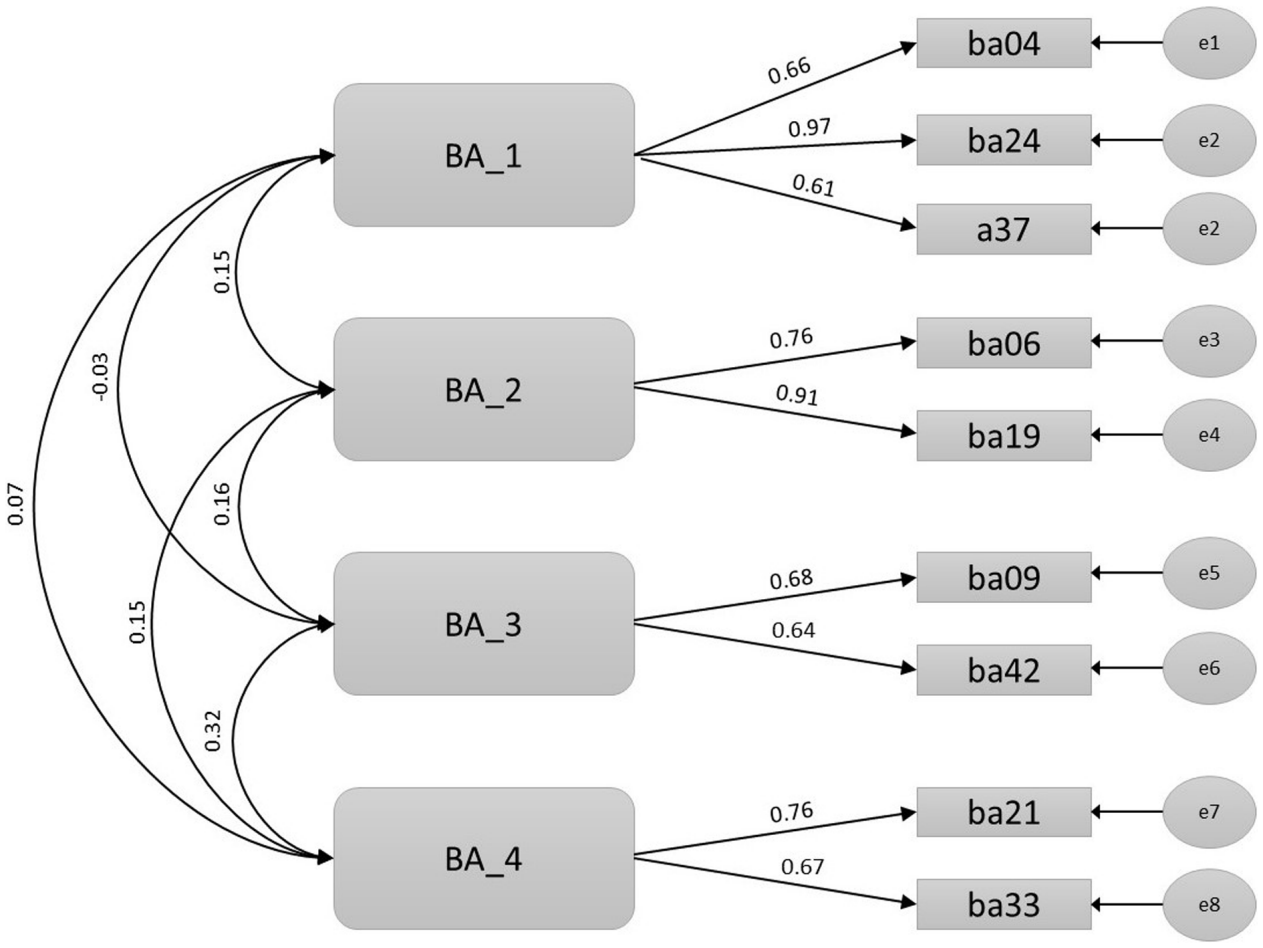
Benefit factors’ measurement model.

**Figure 2:**
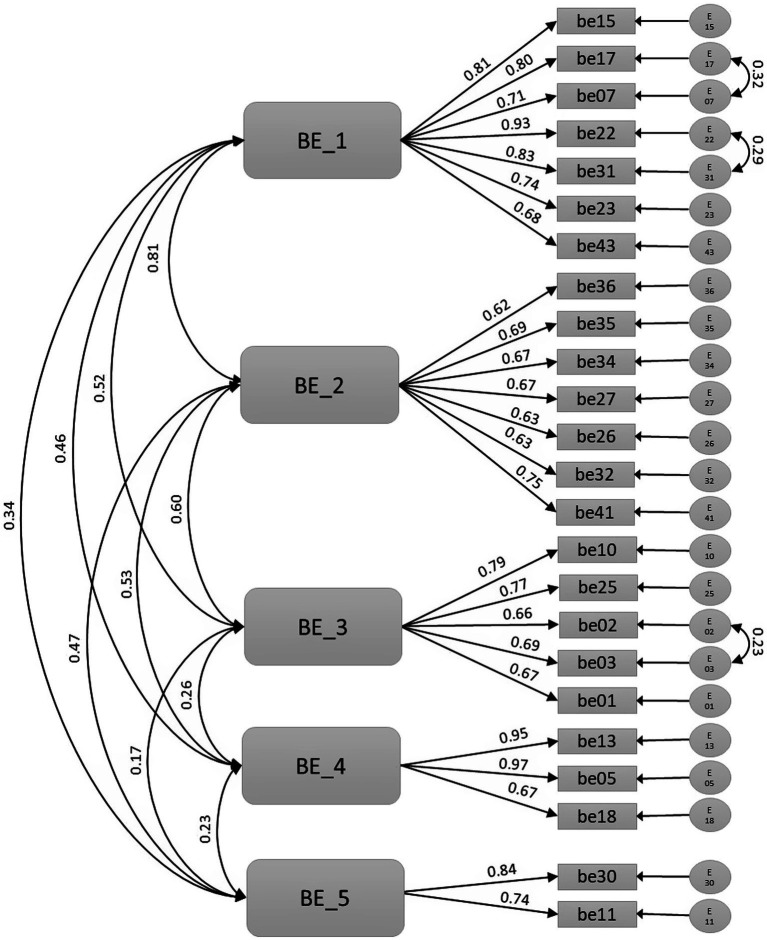
Barrier factors’ measurement model.

In the confirmatory stage, two separated measurement models were tested (Figures 1, 2), and goodness-of-fit indices were calculated in the second half of the sample. Goodness-fit-indexes in both benefits (RMSEA = 0.064, TLI = 0.914, CFI = 0.926) and barriers (RMSEA = 0.061, TLI = 0.936, CFI = 0.961) measurement models were adequate.

Almost all benefit factors are shown to be strongly and statistically significantly correlated (Table 4), but factors of cardiovascular, relaxation, and psycho-social capacities are less related among them. The barriers, on the other hand, have very little correlation among themselves, they are almost independent from each other, although with small positive correlations. There are little or no correlations between barriers and benefits. There is certain association between having less time available and valuing social contact and the most fatigued value relaxation less highly.

**Table 4:** Correlation matrix between benefit and barrier factors.

	BE1	BE2	BE3	BE4	BE5	BA1	BA2	BA3
BE1—Physical								
BE2—Psycho-social capacities	0.690^**^							
BE3—Relaxation	0.437^**^	0.513^**^						
BE4—Cardiovascular	0.491^**^	0.506^**^	0.261^**^					
BE5—Social contact	0.321^**^	0.390^**^	0.187^**^	0.178^**^				
BA1—Time	0.023	0.063^**^	–0.120^**^	0.069^**^	0.145^**^			
BA2—Fatigue	0.049^*^	–0.064^**^	–0.135^**^	0.079^**^	–0.048^*^	0.182^**^		
BA3—Facilities	–0.023	–0.077^**^	–0.068^**^	–0.049^*^	–0.081^**^	0.007	0.122^**^	
BA4—Family	–0.046^*^	–0.014	–0.061^**^	–0.016	–0.055^**^	0.079^**^	0.110^**^	0.184^**^

* *Correlation is significant at the 0.05 level (two tailed);*

** *Correlation is significant at the 0.01 level (two tailed)*.

Higher age is associated with higher scores on benefits related to psycho-social, relaxation, and cardiovascular (Table 5). In addition, it is generally associated with a lower incidence of barriers, but with some nuances that can be seen in the averages.

**Table 5:** Benefit and barrier factor comparison among age groups.

	Means				***F***	value of ***p***	*Post hoc* (Scheffé test with α = 0.10)
	≤25	26–35	36–50	>50			
BE1	5.67	5.68	5.82	5.89	1.416	0.236	-
BE2	4.88	5.05	5.22	5.38	7.237	0.000	36–50 > −25
BE3	6.19	6.42	6.60	6.41	21.003	0.000	26–35 > −25, 36–50 > −25, 36–50 > 26–35
BE4	3.95	4.27	4.83	5.28	25.258	0.000	26–35 > −25, 36–50 > −25, 50+ > −25, 36–50 > 26–35, +50 > 26–35
BE5	4.80	4.61	4.67	5.11	1.817	0.142	
BA1	3.96	4.29	4.27	3.57	6.805	0.000	26–35 > −25, 36–50 > −25, 26–35 > +50
BA2	3.30	3.64	3.27	3.03	5.017	0.002	26–35 > −25, 26–35 > 36–50
BA3	3.92	3.10	3.03	3.12	35.512	0.000	26–35 > −25, 36–50 > −25, 50+ > −25,
BA4	3.12	3.30	2.96	2.35	3.337	0.019	+50 > 26–35

When considering the type of horseback riders (Table 6) professional riders report a higher level of physical benefit and psycho-social capacities, whereas amateur riders show higher levels of relaxation-related benefits. In terms of barriers, professional riders complain more about lack of time, while amateur riders are more concerned about lack of resources and family-related problems.

**Table 6:** Benefit and barrier factor comparison between amateur and professional horseback riders.

	Means		***t***	value of ***p***
	Amateur	Professional		
BE1—Physical	5.64	5.80	-3.491	0.000
BE2—Psycho-social capacities	4.90	5.04	-2.683	0.007
BE3—Relaxation	6.31	6.11	5.361	0.000
BE4—Cardiovascular	4.05	4.14	-1.183	0.237
BE5—Social contact	4.66	5.07	−5.704	0.000
BA1—Time	3.70	4.86	−18.307	0.000
BA2—Fatigue	3.35	3.30	0.752	0.452
BA3—Facilities	3.79	3.62	2.312	0.021
BA4—Family	3.18	2.95	2.862	0.004

Gender differences are statistically significant in three factors (Table 7): Male riders value more social contact compared to female riders, and female riders report higher levels of barriers in facilities and family.

**Table 7:** Benefit and barrier factor comparison between male and female horseback riders.

	Means		***t***	*p*
	Male	Female		
BE1—Physical	5.52	5.70	−1.384	0.169
BE2—Psycho-social capacities	4.97	4.93	0.311	0.756
BE3—Relaxation	6.21	6.25	−0.455	0.650
BE4—Cardiovascular	4.18	4.07	0.699	0.485
BE5—Social contact	5.33	4.75	3.445	0.001
BA1—Time	4.09	4.02	0.452	0.651
BA2—Fatigue	3.15	3.34	−1.318	0.188
BA3—Facilities	3.20	3.77	−3.361	0.001
BA4—Family	2.73	3.13	−2.204	0.028

## Discussion

The aim of the study was to describe and analyze the perceived benefits and barriers in horseback riding among riders categorized by level and age, in order to promote physical activity through these benefits and to overcome the barriers. There is insufficient scientific evidence about the benefits and perceived barriers to horseback riding (Malchrowicz-Mośko et al., 2020), so the results of this study are intended to provide updated information on this issue. Considering the results obtained with the entire sample (*n* = 2,651), the dimensions of the benefits are ranked as follows: psycho-social capacities, relaxation, social contact, physical performance, and preventive health. Comparing these results with previous studies that examined perceived benefits shows both, similarities and differences, with some studies suggesting that the perceived benefits are those of physical activity itself, while in others general health, physical appearance and mental health were the most valued benefits (Ebben and Brudzynski, 2008; Sáez et al., 2021). Regarding the barriers, in our study, they are hierarchically organized as follows: time investment for exercise, lack of facilities, fatigue, and family discouragement. Comparing these results with previous studies, the main barriers affecting the practice of exercise were economic cost, tiredness, and fatigue (Lovell et al., 2010), while in other studies were lack of time and social pressure (Muzindutsi et al., 2014; Blake et al., 2017).

When we drop the entire sample and segment it by different categories we should highlight at different ages the benefits and barriers are perceived differently. The older the participant (≥25 years) the higher the perceived social contact, relaxation, and physical performance are perceived with higher intensity and with statistically significant differences than the younger participants (≤25 years) do, in line with previous research (Lunn, 2010; Belanger et al., 2011). On the other hand, regarding the perception of barriers, lack of facilities is the barrier perceived with higher intensity by the younger participants, unlike other disciplines (Nies and Chrusical, 2002; Rodríguez et al., 2009), due to the characteristics of horseback riding. The rest of the barriers are perceived with similar intensity, as in other studies (Juarbe et al., 2002; Lovell et al., 2010).

Secondly, segmenting the sample between professional and recreational riders, both groups perceive benefits and barriers differently. On the one hand, professional riders perceive greater benefits in terms of physical performance and social contact as in other sports disciplines (Palermi et al., 2020) and unlike other areas, in which the perceived benefits are as: physical performance and preventive health (Oja et al., 2015). In terms of barriers, this group perceives the time investment for exercise as the greatest barrier to the practice of horseback riding. On the other hand, recreational riders perceive psycho-social capacities as the greatest benefit of horseback riding, as in other disciplines (Woods, 2019). Regarding the perceived barriers to continuing horseback riding, lack of facilities and family discouragement were those perceived with higher intensity, accordance with previous research (Mayolas-Pi et al., 2017; Lukács et al., 2019).

Finally, when dividing the sample by gender, it is important to mention that equestrian sport is a female-dominated sport (Burbage and Cameron, 2018) and this is evident in the distribution of the sample in our study. Regarding the perceived benefits of horseback riding, there are no significant differences in four of the five dimensions, possibly conditioned by the differences in sample size, since in other sports disciplines there are significant differences (Speck and Harrell, 2003; Vlachopoulos et al., 2013; Glavin et al., 2021). Only social contact is rated higher among men, as in other studies (Craft et al., 2014; van Uffelen et al., 2017; Blanco et al., 2019). The barriers to horseback riding participation both, men and women, said that time investment for exercise and physical effort are the barriers to horseback riding participation as stated in earlier research (Sequeira et al., 2011; Hosseini et al., 2017; Hurley et al., 2018). In addition, similar significant differences were found in research analyzing the difference between genders (Rosselli et al., 2020; Ghorbani et al., 2021) in lack of facilities and family discouragement. To conclude the discussion through the gender difference, to the barriers analyzed by the EBBS, it would be interesting as has been analyzed in other research to add the barrier of anatomical characteristics (e.g., influence of the chest) as a barrier to horseback riding (Burbage and Cameron, 2018).

## Conclusion

This study aims to improve the understanding of perceived benefits and barriers among horse-riding athletes, focusing more on differences in age and competitive level. In particular, it was the older participants who most strongly perceived benefits in social interaction, psychological outlook, and physical performance. However, younger participants perceived barriers more strongly than older athletes.

The main factors in terms of benefits, among athletes of different competitive levels (professional vs. recreational), were perceived with higher intensity in physical performance and social contact among professional athletes and relaxation among recreational athletes. In addition, regarding the perception of barriers, professionals perceived time investment for exercise as the greatest barrier and exercise-related environment and family discouragement among recreational athletes.

Therefore, these results can contribute to the promotion of horseback riding as an opportunity to comply with the recommendations of international organizations to be considered active people. Equestrianism is a discipline in which the perception of benefits is higher than that of the barriers to its practice. On the other hand, it would be interesting to minimize the barriers and promote and visualize the benefits (physical, social, and psychological) as possible points of action to promote health through horseback riding.

Some limitations must be assumed in the present study. The main limitation is in the decompensation of the sample with a higher number of women than men and the difference between professional and recreational riders. This can be explained because as in any other discipline, the number of professional practitioners is significantly lower than that of recreational ones and as Burbage and Cameron (2018) say horseback riding is a sport with a higher number of female riders.

## Data Availability Statement

The raw data supporting the conclusions of this article will be made available by the authors, without undue reservation.

## Ethics Statement

Ethical review and approval was not required for the study on human participants in accordance with the local legislation and institutional requirements. Written informed consent for participation was not required for this study in accordance with the national legislation and the institutional requirements. The research was carried out in accordance with the Declaration of Helsinki.

## Author Contributions

IS, PL-G, and EM-M: conceptualization, methodology, and investigation. EM-M: validation and data curation. JS, IS, and EB: formal analysis and resources. IS, BL, XR, and EB: writing—original draft preparation. IS, JS, BL, XR, PL-G, and EB: writing—review and editing. All authors contributed to the article and approved the submitted version.

## Conflict of Interest

The authors declare that the research was conducted in the absence of any commercial or financial relationships that could be construed as a potential conflict of interest.

## Publisher’s Note

All claims expressed in this article are solely those of the authors and do not necessarily represent those of their affiliated organizations, or those of the publisher, the editors and the reviewers. Any product that may be evaluated in this article, or claim that may be made by its manufacturer, is not guaranteed or endorsed by the publisher.
